# Supplementary material for: Health Recommender Systems Development, Usage, and Evaluation from 2010 to 2022: A Scoping Review
Source: Int J Environ Res Public Health. 2022 Nov 16;19(22):15115. doi: 10.3390/ijerph192215115 (PMC9690602; doi:10.3390/ijerph192215115)
Supplement: Supplementary file 1 [file ijerph-19-15115-s001.zip › Supplement 1.pdf]

Supplement S1

Table S1. Search strategy of ACM Digital Library.

Search number	Search term
1	"recommendation system" OR "recommendation systems" OR "recommender system" OR "recommendersystems" OR personalize OR personalization OR personalized
2	health OR healthcare OR patient OR patients
3	#1 AND #2

Results: 545

Table S2. Search strategy of IEEE Xplore.

Search number	Search term
1	All Metadata: "recommendation system" OR "recommendation systems" OR "recommender system" OR "recommender systems" OR personalize OR personalization OR personalized
2	All Metadata: health OR patient OR healthcare OR patients
3	#1 AND #2

Results: 3,061

Table S3. Search strategy of PubMed.

Search number	Search term
1	Title/Abstract: "recommendation system" OR "recommendation systems" OR "recommender system" OR "recommender systems" OR personalize OR personalization OR personalized
2	Title/Abstract: health OR patient OR healthcare OR patients
3	Title/Abstract: information OR ”self management” OR ”decision making” OR ”social support” OR "health behavior” OR education OR ”self care” OR empower OR ”well being
4	Title/Abstract: medicine OR genes OR genetic OR cellular OR genomic OR molecular OR protein OR cell
5	#1 AND #2 AND #3 NOT# 4

Results: 5,647

Table S4. Search strategy of Scopus.

Search	Search term

number	
1	TITLE-ABS-KEY: "recommendation system" OR "recommendation systems" OR "recommender system" OR "recommender systems" OR personalize OR personalization OR personalized
2	TITLE-ABS-KEY: health OR patient OR healthcare OR patients
3	TITLE-ABS-KEY: information OR ”self management” OR ”decision making” OR ”social support” OR "health behavior” OR education OR ”self care” OR empower OR ”well being
4	TITLE-ABS-KEY: medicine OR genes OR genetic OR cellular OR genomic OR molecular OR protein OR cell
5	#1 AND #2 AND #3 NOT# 4

Results: 5,614

Table S5. Searching strategy of Web of Science.

Search number	Search term
1	"recommendation system" OR "recommendation systems" OR "recommender system" OR "recommender systems" OR personalize OR personalization OR personalized
2	health OR patient OR healthcare OR patients
3	information OR ”self management” OR ”decision making” OR ”social support” OR "health behavior” OR education OR ”self care” OR empower OR ”well being”
4	medicine OR genes OR genetic OR cellular OR genomic OR molecular OR protein OR cell
5	#1 AND #2 AND #3 NOT# 4

Results, 5,388
