# Supplementary material for: Health Recommender Systems Development, Usage, and Evaluation from 2010 to 2022: A Scoping Review
Source: Int J Environ Res Public Health. 2022 Nov 16;19(22):15115. doi: 10.3390/ijerph192215115 (PMC9690602; doi:10.3390/ijerph192215115)
Supplement: Supplementary file 1 [file ijerph-19-15115-s001.zip › Supplement 2.pdf]

Supplement S2

Table S6 Summary of health recommender systems.

Article	Health domain	User		Recommended item		Recommendation technology			System evaluation	
		Target user	User model	Recommended information	Information source	Algorithms	System name	User interface	Method	Measurement/ metrics
[57]	Sexual and reproductive health disease	Woman with different degrees of hearing loss	Demographic data, medical data, implicit data	Sexual and reproductive health content	Doctors, content producers, educators and sign language interpreters worked together in content design and preparation	Hybrid (Probabilistic Graphical Models + Rule based reasoning)	MCS	Web-based (http://mesade Org/mcs/)	Experiments conducted with 22 students with a bachelor's degree and 16 students with a master's degree	Volunteers filled in a questionnaire (Quality of the recommendation contents, importance to offer content through recommender system)
[31]	Low back pain	Patients with nonspecific low back pain	Demographic data, medical data, physical activity	Physical activity, education, strength and flexibility exercises	Existing knowledge (clinical guidelines and medical ontologies) and information provided by	Case based reasoning	selfBACK	Mobile-based	Multinational, randomized controlled experiment, targeting care-seeking patients with nonspecific low back pain,	A process evaluation to document the implementation and patient experiences with selfBACK

					patients				comparator will be patients who receive the usual treatment	
[60]	Functional somatic symptoms (FSS)	Patients with mild to moderate FSS	Explicit data	Self-help Exercise	Offered by experienced clinicians	The algorithm was produced based on the input of clinicians who experienced in FSS treatments.	Grip self-help	Web-based (http://www.nedap-healthcare.com/)	A pilot study	Symptom severity, health related quality of life, productivity loss, healthcare utilization and costs, acceptability to both patients and primary care professionals
[59]	Schizophrenia	Patients with schizophrenia	Medical data	Advice, suggestions, tips and information	Information from evidence-based research, clinical	Problem severities algorithm	Wegweis	Web- based	(1) Compared the problem severity that system generated with clinicians’	Precision, recall, F-measure

					expertise, and patient experiences				opinions; (2) patients evaluated the relevance of system selected topics	
[23]	Asthma	Patients with asthma	1.Explicit 2.Medical data	1.Educational resources 2.Behavioral procedures, relaxation training, asthma self-management outside home	1.Research papers and presentations 2.Ontology design relied on health professionals in asthma treatment and ontologies experts	1.[a]CF 2.Rule based	Astmapp	Mobile-based	Thirty asthma patients used Astmapp: 1. patients selected relevant resource; 2 experts determined whether recommendation was correct or not	1.Effectiveness (precision, recall, F-measure) 2.precision & recall
[62]	Heart disease	Patients with heart disease	Demographic data, Medical data	Medical recommendation	Created by physicians	Rule based			Clinical experts verified the accuracy of the recommendation model	Confusion matrix

[61]	Sleep	General public	Demographic data, Medical data, Physical activity, Explicit data	Texts about "Consistency, winding down, exercise, duration"	General sleep hygiene guidelines	CF			Cohort-based recommendation compared with either "no-recommendations" or general recommendations scenarios	Sleep time, time to fall asleep, number of awakening per hour, subjective sleep quality, Epworth Sleeping Scale (ESS) score, Pittsburgh Sleep Quality Index (PSQI) score
[58]	Elderly care	Elderly persons	Demographic data, Medical data, Food intake, Physical activity	Physical therapy, food, emotional support, exercise		Case based reasoning	An Elderly Care Recommendation System	Web-based	Five elderly persons participated in system testing and the results were evaluated by three experts.	Each expert evaluated the recommendation solution and calculated the percentage of correct solutions.
[74]	Stress	General	Medical	Message	18 stress	Q-	JITAI (just-	Mobile-based	Compared the	Number of

		public	data	downloaded from google play store	micro-interventions	learning	in-time adaptive intervention)		performance of Q-learning policy with random policy	interventions to relieve stress
[34]	Traditional herbal medicines	General public	Demographic data, Medical data	Traditional herbal medicines	Three knowledge sources were integrated to construct a traditional herbal medicine ontology*	Rule based			27 patients participated in three testing scenarios and the system recommendation results were assessed by a medical specialist.	Accuracy, precision, recall, F-measure
[35]	Drugs	Patients with hypertension	Demographic data, Medical data	Anti-hypertensive drugs	Guidance from cardiovascular experts and "Chinese guidelines for management of hypertension"	Rule based			System performance results benchmarked by physician prescriptions	"upgrade rate of ranking"
[27]	Cancer	Cancer patients	Demographic data, Medical	A daily diet menu with a list of		Hybrid (Case based +			Use case study	Usefulness and accuracy

			data, Physical activity, Explicit data	recommended dishes		rule based + genetic algorithm)				
[28]	Cancer	Patients with breast cancer	Medical data, Explicit data	Health information related to breast cancer	Health information vetted by healthcare professional*		MyPath	Mobile-based	Seven-month deployment study	Usability analysis using usage logs and participants' interviews
[29]	Cancer	Patients with lung cancer	Demographic data, Explicit data	Medical articles		Hybrid (Content-bootstrapped CF)			Empirical study comparing algorithm performance with CF and [b]CB	Mean absolute error, receiver operating characteristics
[44]	Depression	Patients with depressive symptoms	Explicit data	Activity recommendation	384 pleasant activities was created by combing activities from the Pleasant Event Schedule	CB	MUBS	Mobile-based	8-week feasibility study with 17 patients with depression	Semi-structured interviews on usage of the app; questionnaire on future acceptance

					Work of other researchers.					
[45]	Depression	General public	Demographic data, Medical data, Physical activity, Explicit data, Implicit data	Activity recommendation	Emotion regulation action knowledge based on experts	Hybrid (clustering + conditional combinations cube)	Emotion-regulation recommender system	Mobile-based	Proposed method compared with random and conditional combination cube methods	Mean average position, mean absolute error
[40]	Overweight and obesity	General public	Medical data	Healthy food restaurants	Healthy food restaurant information available in Internet	CF	H-Rec[2]	Mobile-based	The proposed algorithm compared with three other approaches: random, ItemCF-based-user interest history, ItemCF-based-user interest and health level	Participants' scores to the recommendations, user's experience and intention

[41]	Obesity	General public	Demographic data, Physical activity	Calorie intake		Rule based	Idiet	Mobile-based	An example illustrated how the algorithm works.	Comparing recommendation results with expected theoretical values
[42]	Obesity	Youth with obesity	Demographic data, Medical data, Food intake	Dietary nutrition menu	Dietary nutrition database from 132 obese youth who succeed in reducing and controlling their weight	Hybrid (Knowledge based + CF)	Dietary Nutrition Recommendation for Obese Management Service	Mobile-based	The proposed algorithm was compared with CF; 100 obese youth were surveyed for their app use and satisfaction.	Precision, recall, F-measure, survey results
[51]	Diabetes	American Indian (AI) diabetic patients	Demographic data, Medical data, Explicit data, Implicit data	Guideline (i.e., Diabetes general recommendations, food and nutrition, physical workout, AI-related healthcare)	Authoritative public documents and websites*	Rule based	MobiDiaBTs	Mobile-based	User case studies and human experts' verification	Accuracy, relevance, appropriateness
[41]	Diabetes or	Patients	Demograp	Education		Rule		Web-based	Use case	

	obesity	with diabetes or obesity	hic data, Medical data, Physical activity, Explicit data	content		based reasoning			scenario	
[52]	Diabetes	Patients with Type 2 diabetes	Medical data, Food intake	Diet recommendation	USDA food composition database	Rule based			Compared the system performance with clinical narratives and gold standards developed by clinicians	Consistency with clinical narratives and gold standards
[53]	Diabetes	Patients with Type 1 diabetes mellitus	Medical data, Food intake, Physical activity	Bolus insulin recommendation		Case based reasoning			The algorithm performance compared with default insulin therapy, R2R method, and Herrero's proposed method during virtual testing with 33	Time in, above, and under the target glycaemia range; system robustness; & system latency

									subjects	
[54]	Diabetes	Patients with diabetes	Demographic data, Medical data, Food intake, Physical activity	Messages about diabetes control and health habits	Predefined recommendations by a medical team	Case based reasoning	glUCModel	Web-based		
[45]	Diabetes	Patients with Type 1 diabetes	Demographic data, Medical data, Physical activity	Carbohydrate intake and insulin dosage before, during and after exercise		Case based reasoning			10 Type 1 diabetic patients evaluated the usability of the system	usability feedback
[24]	Arterial hypertension	Patients with hypertension	Demographic data, Medical data, Physical activity, Implicit)	Physical activity		CF			Validated by 9 physicians	Recommendation approved rate through a questionnaire
[25]	Hypertension	Patients with hypertensive	Demographic data, Medical data, Explicit	Mauritian diet plans	Weight-Less book, sodium of food from the USDA food	CB	DASH	Mobile-based	10 hypertensive patients used the app and completed a	User feedback (eating requirements, control and reduce blood

			data, Food intake		composition database				post-test survey questionnaire.	pressure level)
[33]	Cardiovascular disease	Patients with cardiovascular disease	Demographic data, Medical data, Food intake, Physical activity, Explicit data	Educational material	Message library *	Rule based	PULSE (Personalized Using Linkages of Score and behavior change readiness to web-based Education)	Web- based	Survey based Pilot user study involving cardiovascular disease patients	Willingness to follow suggestions offered by PULSE, information quality
[36]	Chronic diseases	General public and patients with diet-related chronic diseases	Medical data	Calabrian food	Criteria certified by the medical team as well as the nutritional information compiled by nutrition specialists	Health-related, diseases-related, and food-related	DIETOS (DIET-Organizer System)	Web-based (http://www.easyanalysis.it/dietos)	Compared with other diet-related chronic disease recommender systems	Analytical comparison of methodologies, user categories, & clinical experimentation
[37]	Chronic diseases	General public and patients	Demographic data, Medical	Diet recommendation	Taiwanese snacks Nutrition	Rule based			15 users participated in the experiment	Accuracy

		suffering from diabetes, hypertension, & high cholesterol	data, Physical activity, Explicit data		Analysis				and nutritionist evaluated the system performance	
[38]	Chronic diseases	Patients with chronic diseases	Demographic data, Medical data, Physical activity, Food intake, Explicit data	Dietary recommendation	Taiwan food nutrition database of the Food and Drug Administration	Rule based			Recommendation results are evaluated by dietitians.	Accuracy
[39]	Chronic disease	Patients with chronic disease	Demographic data, Medical data, Food intake, Physical activity	Educational materials	Websites, guidelines, and books, reviewed and approved by physicians*	Rule based			Experts scored each document based on the feedback from 50 patients.	Macro precision of top-ranked documents, overall mean average precision
[66]	Health service	General public	Demographic data, Medical	Family doctor	Doctor registration data obtained	Hybrid (CB + CF) with			The algorithm compared with heuristic	Analytical evaluation by hit rate and

			data, Implicit data		from Human resources department	quantitative trust measure			baseline, CF, CF-trust, & hybrid	precision
[67]	Health service	General public	Implicit data	Health service (symptom, disease, department, doctor)	Collected information by crowdsourcing	Case based reasoning			eHeaRSS compared with general DB-based health systems	Patients' satisfactions
[68]	Health service	General public	Demographic data, Medical data, Explicit data	Doctor recommendation	Registered doctors	Hybrid (CB + CF + demographic filtering)	Medicare	Web-based	9 doctors and 8 patients participated in system quality evaluation	Precision, recall, F1, mean absolute error
[69]	Health service	General public	Demographic data, Medical data, Explicit data	Doctors and hospitals		CF		Web-based	Only user cases provided	
[48]	Online health forum	General public	Explicit data, Implicit data	Health forum Thread recommendation	Health forum thread	Interest aware topic model (IATM) +			Compared with CF, [c]AT, [d]CTR, IATM, [e]CAR, AT+JNCTR	Recall, mean reciprocal rank, normalized discounted

						jointly normalized collaborative topic regression (JNCTR)			on two real world consumer health forums: PatientsLikeMe and HealthBoards	cumulative gain
[49]	Online health communities (OHC)	General public	Implicit data	Discussion threads recommendation	OHC threads	Multilayer perceptron in an interest matching neural network			Compared with the [f]TR-XMLC, [g]CVAE on “cancer survivor network”, MedHelp, and HealthBoards	Recall, normalized discounted cumulative gain, mean reciprocal rank
[50]	healthcare social media	General public	Implicit data	Similar user	Health social website MedHelp: user, drugs, disease, [h]ADRs*	Hybrid (CB + structural approach)			Compared with CB approach	F1 score, precision, recall
[75]	Promoting active lifestyle	General public	Demographic data, Medical data,	Food, mental therapy, physical therapy		Hybrid (rule based + probabilist	ATHENA (activity-awareness for human-	Web-based	Case scenarios comparison with naïve bayes, random	Accuracy, F-score

			Physical activities, Food intake, Explicit data			ic model + case based)	engaged wellness application)		forest, 1-Nearest Neighbor	
[76]	Healthy diet	General public	Demographic data, Medical data, Food intake, Explicit data	Dietary Information	[j]USDA database to collect food ingredients and nutritional data	Rule based	DISYS	Web-based	Only typical usage scenarios provided	
[77]	Nutrient balance	General public	Food intake	Foods for meals-out		Detecting foods for the target nutrients balance			Compared with other algorithms	Amount of Intake Energy, Amount of protein, fat, and carbohydrate intake
[78]	Balanced and healthy diet	General public, patients with diabetes	Medical data, Food intake, Explicit data	Recipe recommendation	Recipes from reliable source*	Rule based	DRS	Web-based	Only user cases provided	

		mellitus, or patients with hypertension								
[79]	Health lifestyle	General public	Food intake, Physical activity	Food and activity	Users' past physical activities and food intake	Multi-armed bandit	Mybehavior	Mobile-based	A 3-week, two-group controlled experiment	Quantitative and qualitative comparison (walking lengths, food calories, and user technology acceptance, & suggestions)
[80]	Physical activity	Adult with a low risk for acute cardiovascular during physical activity and a sedentary lifestyle	Medical data, Physical activity	Daily Physical activity prescription	American College of Sports Medicine Training Progression Guidelines	Rule based	PPAP (personalized physical activity prescription)	Web-based	Pilot study (two subjects during 12 weeks of physical activity training)	Mean training impulse (calculated by weekly recommended and completed physical activity volume)

[66]	Prolonged inactivity	Prolonged sitting/ standing/ lying down public	Demographic data, Medical data, Physical activity	Physical activity messages, educational facts	Sedentary behavior guidelines	Rule based	Mining Minds	Web-based	10 volunteers with 40 different types of sedentary behaviors used the app for 2 weeks; system performance was compared with a baseline	Average execution time and accuracy
[82]	Healthy lifestyle	General public	Demographic data, Medical data, Food intake, Physical activities, Explicit data	Diet and physical activity		Particle swarm optimization based algorithm			Evaluated using a set of system-generated user profiles	
[63]	Smoking cessation	Smoker	Demographic data, Medical data, Explicit data	Motivational messages for staying on smoking-free.	Researcher specializing in behavior change theories designed messages	Hybrid (Knowledge-based + demographic filtering)	Quit and Return	Mobile-based	Planned feasibility study of 1,050 smokers	[K]Planned metrics for system evaluation

[64]	Smoking cessation	Smoker	Demographic data, Medical data, Explicit data, Implicit data	General motivation, diet tips, physical exercise, personal performance, benefits of non-smoker health messages	messages approved by a smoking cessation psychologists and a pulmonologist from the hospital	Patient's demographic similarity, perceived utility of the message topics, statement of initial interest	Smokefree	Mobile-based	120 patients' feedback on messages and their interactions with the app	[L]Objective quality and subjective quality along with engagement metrics
[65]	Smoking cessation	smoker	Explicit data, Implicit data	Motivational message	messages produced by both experts and peers; messages informed by current guidelines and social cognitive theory	Hybrid (CB + CF)			Compared with a standard computer tailored health communication system in a prior study	Daily message rating, intervention's perceived influence, 30 days cession, changes in readiness to quite from a baseline
[46]	Mental health	General public	Explicit data	Intervention text	Messages produced by behavior	Hybrid	PAX	Mobile based	---	---

					change theories					
[83]	Covid 19	Covid 19 patient	medical data, explicit data	Patient care plans	Integrating the web services of the relevant caregivers and service providers into an executable workflow	Knowledge based	---	Web based	---	---
[47]	Mental health	General public	Explicit data, implicit data	New therapy tasks	76 different options from categories of “basics”, “fitness”, “fun”, “social”, “art” and other	Collaborative filtering	---	---	Compared with random and a simpler baseline algorithm	Mean absolute error (MAE), root mean squared error (RMSE)
[70]	Health service	General public	Medical data, explicit data	Hospitals and doctors	Hospitals gathered from various websites	Hybrid	HealthFinder	Web based	Patients feedback	Rating the recommended items
[71]	Health services	General public	Explicit data	Doctors	Doctors gathered from website	Hybrid	---	---	Compared with those based on only	---

									similar patients and only based on similar doctors	
[26]	Hypertension	Patients with high blood pressure	Medical data, physical activity	Lifestyle (physical activity, sleep)	---	Random forest with feature selection	---	---	Compared with 25 patients with elevated BP or stage I hypertension not receive lifestyle recommendations	Change of BP levels
[84]	Alzheimer	Patients with Alzheimer's disease	Demographic data, medical data	Home care	Medical articles, encyclopedias, online websites, clinical experts	Rule based	---	---	Evaluated by domain expert	Computational efficiency, completeness, consistency, conciseness
[85]	Hemophilia	Patients diagnosed with hemophilia	Medical data, physical activity,	Health resources, educational materials	Scientific literature, public health agency	Knowledge based	HemPHL	Mobile based	A panel of subject matter experts assess the HemPHL	Patient's knowledge on hemophilia, clinical

			implicit data		websites					outcomes, health-related quality of life, satisfaction with PHL platform
[32]	Atrial fibrillation, lower back pain	patients	Demographic data, medical data	Educational materials	Clinical vocabulary, HL7 fast healthcare interoperability resources (FHIR)	Knowledge based	---	Mobile based	End users evaluated the usability of the system	Perceived ease of use, perceived usefulness, perceived intention to use, recommendation
[72]	Health service	General public	Medical data	Doctor	---	Probability based classifier and decision tree based classifiers	---	---	Compared with C4.5 and Naïve bayes classifier	Accuracy and F1 measure
[30]	cancer	Patients with cancer	Medical data, physical activity,	Therapeutic exercise	Experts modified recommendations and new	Knowledge based	ATOPE+	Mobile based	Tested with patients with breast cancer, and evaluated	Usability

			explicit data		rules				by clinical experts	
[73]	Health service	General public	Explicit data	Hospital	---	Collaborative filtering	Health Monitoring	Web based	---	---
[56]	Diabetes mellitus	Patients with diabetes	Medical data, physical activity	Diet menu	American diabetes association	Knowledge based	MANFIS	Web based	Ten selected experts evaluate the system	Accuracy, clarity, understandability, user interface friendliness

Abbreviation

[a]CF: collaborative filtering

[b]CB: content-based

[c]AT: author-topic model

[d]CTR: collaborative topic regression

[e]CAR: context aware recommendation

[f]TR-XMLC: Thread recommendation-eXtreme Multi-Label Classification

[g]CVAE: collaborative variational autoencoder system

[h]ADRs: averse drug reactions

[j]USDA: United States Department of Agriculture

[K] Planned metrics for performance assessment: smoking cessation rate, days before relapse, user engagement at an individual level, smoking abstinence, quality adjusted life years (financial aspects), precision of recommender system, user engagement at an aggregated level, user reliability, user app behavior, user quit attempts, user

satisfaction with message, user mobile app usage, user message ratings.

[L] Objective quality: precision, time to read;

subjective quality: questionnaire intend to measure the quality of user's experience with the recommender system and its influence on the user's behaviors and intentions, whether the messages have an impact on the patients' motivations;

patients' engagement: rolling retention, session length distribution, session frequency, session per user, return rate, individual engagement.

Note: Studies with * specified individual information source they used, and they are listed in Table 10 below.

Table S7 Information source clearly specified by some studies.

Article	Information source
[34]	(1) Notification of the National Drug System Development Committee on the National List of Essential Medicines 2018;<br>(2) Classification of Diseases, Symptoms, and Procedures of Thai Traditional Medicine, Version 2015;<br>(3) ICD-10-TM: International Classification of Diseases (Thai Modification), Version 2016
[28]	American Cancer Society;<br>breastcancer.org, cancer.net;<br>cancer navigation;<br>cancer clinic websites.
[51]	General guideline from: American diabetes association (ADA), the British dietetic association (BDA), Association of Clinical Endocrinologists and American college endocrinology (AACE/ACE);<br>Nutrition guideline from Dietary Guidelines for American (USDA) & Dietary Reference Intake.<br>Physical guideline from American Diabetes Association (ADA), Eat Heathy – Be Active Community Workshops (EHBA) and American Heart Association;<br>Healthcare guidelines from: Indian Health Services (IHS), American Indian and Alaska Health, & National Indian Health Board.
[33]	"the Healthy Heart Kit" from public health agency;<br>Cardiovascular & Pulmonary Health in Motion Cardiac Rehabilitation Program;<br>Heart & Stoke Foundation website;

	Health Canada's "food guide to healthy eating" ;<br>"physical activity guide to healthy active living "
[39]	Websites: https://dxy.com/column; http://www.39.net/,<br>Guidelines: Chinese Guidelines for Prevention and Treatment of Hypertension (2013)， Guidelines for Diabetes Care and Education in China （2010），Guidelines for Exercise Therapy in Diabetes in China（2014），<br>Books: Daily Care and Expert Guideline Full Program of High Blood Pressure（2016），Daily Care and Expert Guideline Full Program of Diabetes（2016），Hypertension（2015）, Diabetes（2015）
[50]	healthcare social website users: user name;<br>Drugs: DrugBank<br>Disease: UMLS Metathesaurus<br>ADRs (adverse drug reactions): side effect resource (SIDER)
[78]	Recipe from two website: http://cookpad.com ; http://marron-dietrecipe.com/category/category.html<br>The health nutrition database was built based on "Food Composition Database (FCD)" and Japan Preventive Association of Life-style related Disease (JPALD),<br>Rule based on recipe, FCD and JPALD
