# Supplementary material for: Health Recommender Systems Development, Usage, and Evaluation from 2010 to 2022: A Scoping Review
Source: Int J Environ Res Public Health. 2022 Nov 16;19(22):15115. doi: 10.3390/ijerph192215115 (PMC9690602; doi:10.3390/ijerph192215115)
Supplement: Supplementary file 1 [file ijerph-19-15115-s001.zip › Supplement 3.pdf]

Supplement S3

Table S8 Summary of user model parameters.

Article	Demographic data	Medical data	Food intake	Physical activity	Explicit data	Implicit data
[57]	Age, instruction level, learning level	Health status (type of disability)				Levels of knowledge preferences and needs (estimated through track of user interaction with the platform)
[31]	Demographics, employment and work characteristics	Health status (current LPB and history of LBP, pain medication use, pain-related disability and function, work ability, physical activity level, comorbidities, quality of life, sleep problems, fear-avoidance beliefs, pain self-efficacy, illness perception, perceived stress, mood，LBP and function, fear-avoidance beliefs, work ability, sleep quality, pain self-efficacy, perceived stress, mood,		Number of steps, inactivity, goal setting for physical activity		

		barrier for self management, adherence and rating of exercises)				
[60]					Assessing perpetuating factors using online questionnaires	
[59]		Health status (schizophrenia-related problems & problem severity)				
[23]		Health status (symptoms, vital signs, triggers, & mood)			Patients’ resources ratings	
[62]	Age, gender, BMI	Lab test (systolic and diastolic blood pressure, chest pain type, chest pain location, fasting blood sugar, oxygen saturation, cholesterol, ECG value, heart rate, and heart rate type);<br>Disease history (family history of diabetes and coronary artery disease, hypertension, and smoking history)				

[61]	Height, weight, gender, BMI	Health status (time to fall asleep, number of wakeups per hour, sleep recovery index, sleep time)		Exercise (average number of days per week they exercised vigorously for 30 minutes or more)	Rate sleep quality	
[58]	Gender, age, blood type, weight, height, religion, marital status, education, occupation, income	Health status (high blood pressure level, heart rate, seeing, listening, teeth, medication, constipation, pressure sores, enteral tube feeding, urinary catheterization, left/right arm/leg weakness, rotate the ankle, dismal, bored);<br>Disease history (Type 1 diabetes, Type 2 diabetes, other diseases, drug allergic, food allergic smoking, alcoholism,);	Exercise, energy drink, salty food, sweet food	Daily activities: bathing, grooming, dressing, toilet use, bowels, bladders, feeding, stairs, mobility, transfer		
[74]		Lab test (Heart rate)				
[34]	Age, gender	Health status (pregnancy, lactation, chronic diseases or other diseases);<br>Lab test (body temperature);<br>Medication (currently used				

		medicine)				
[35]	Age, gender, income, job, body weight, waistline, BMI	Lab test (Body temperature, diastolic pressure, systolic pressure, heart rate, respiratory rate, AB, Clone, eGFR, LVMI);<br>Disease history (smoke, drink)				
[27]	Age, gender, ethnic	Health status (cancer, stage of cancer, treatment, stage of treatment, side effect);<br>Disease history (allergy)		Level of activity	Food preferences	
[28]		Health status (diagnosis, treatment)			National comprehensive cancer network survey to identify patients' needs and challenges (practical problems, family problems, emotional problems, spiritual concerns, physical problems)	
[29]	Demographic data				Four target	

					categories of lung cancer article selection & recommended article rating	
[44]					Ratings provided by patients once they completed activities	
[45]	Age, gender, occupation, office hour	Health status (depression level, anxiety level, stress level)		Active item history (cognitive game, mindfulness practice, sport, music, movie, reading, communication, food)	Favorite activity	Sensor (time, location, weather), mobile app usage
[40]		Health status				
[41]	Age, BMI, gender			Physical activity		
[42]	Gender, age, height, weight, BMI	Health status (disease information)	Daily average calorie intake			
[51]	Demographic information	Health status (vital signs, measurement data, genetic, hearing, speech, vision, reading, disease);<br>Disease history (allergy)			Health insurance provider, medical provider, preference (preferred	Social (online social profile, online social network, online friends, offline

					exercise, preferred food, preferred provider)	social profile)
[41]	Age, gender, ethnicity, education level (readability needs and language preferences)	Health status (symptoms and complications, specific treatments, health checks, more generic treatments)		Activity objectives (current physical activity level)	Preferred activities	
[52]		Lab test (pre- and 2-hour-post-meal blood glucose readings)	Meal photos and brief description, mealtime label, meal ingredients,			
[53]		Lab test (current blood glucose level)	Carbohydrate intake, time of day	Past and future physical activity		
[54]	Name, address, height, birth date, weight	Lab test	Carbohydrates ingested	Type of exercise, intensity, duration		
[45]	Age, weight, weight excess, gender	Health status (other chronic disease);<br>Lab test (stage (before, during, after) blood glucose levels);		Intensity of the physical exercise, session information (exercise type, activity within 90 minutes of short-acting insulin, time of day)		
[24]	Gender, age, height, BMI,	Lab test (abdominal circumference, systolic blood pressure, diastolic blood		Physical activity practice group (physical activity,		Physical activity result index (date, period, PARI

		pressure, cardiovascular, total cholesterol, LDL, HDL, Triglycerides, insulin resistance, diabetes mellitus, stroke, coronary artery disease, cardiac insufficiency, chronic kidney disease, left ventricular hypertrophy, carotid artery plaque, carotid femoral pulse wave velocity, ankle brachial index);<br>Disease history (smoking history)		intensity, amount, frequency)		systolic blood pressure, PARI diastolic blood index, PARI body Mass Index, PARI abdominal circumference)
[25]	Age	Lab test (blood pressure);<br>Disease history (allergies, smoking level, alcohol level)	Dietary intake		Preference	
[33]	Age, gender	Health status (depression, stress, cardiovascular disease risk level);<br>Lab test (blood pressure, lipid profile, Glycemic control, weight control);<br>Disease history (health problems, family history, smoking, alcohol)	Dietary habits	Exercise	Staged risk factor (behavior information, stage of change)	
[36]		Questionnaires prepared by				

		the medical specialists to obtain user health profile				
[37]	Height, weight	Health status (kidney function, hypertension, hyperlipidemia)		Activity levels	Food preference	
[38]	Age, sex, height, weight, hip, waist	Health status (diseases);<br>Lab test (blood pressure)	Diet record of past seven days.	Activity levels	Dietary preferences	
[39]	Sex, BMI	Health status (Minimal/mild/ moderate/ moderately severe/severe depression, pregnancy);<br>Lab test (Blood glucose, total cholesterol, triglyceride, high/low density lipoprotein, uric acid, blood pressure);<br>Disease history (Hypertension, diabetes, stork, hyperlipidemia, coronary artery disease, chronic obstructive pulmonary disease, other disease, smoking, drinking);<br>Medication (antihypertensive drugs, hypoglycemic drugs, hypolipidemic drugs)	Diet	High/moderate/low activity level		
[66]	Demographic data	Health status (International				Interaction data,

		Classification Diseases (ICD-9) code)				behaviors data
[67]						Patients' symptoms, diseases, departments, & doctors
[68]	Location, age, gender, average charge	Health status (diseases, severity)			Patients' favorite doctor & doctor rating	
[69]	Name, gender, age, marital status, blood group	Disease history (surgical history)			Ratings on doctors and hospitals	
[48]					User subscribed health forums	User-document interaction
[49]						Users' participation in threads used to capture user interest
[50]						Consumers' posts and participation in discussion threads
[75]	Gender, age, height, weight	Health status (sleep, mental activity)	Food intake (fast food, snacks, slow	Physical activity (walking, jogging,	Preferred (food, activity, music,	

			food)	running, & cycling, rest)	social media)	
[76]	Demographic data	Health status	Dietary		Preference for ingredients	
[77]			Users' food logs on last two parts (user ID, restaurant name, name of the food, size, date and time when the user had/ logged, the amount of energy/ protein/ fat/ carbohydrate)			
[78]		Health status	Dietary record		Preference	
[79]			Dietary intake	Physical activity		
[80]		Lab test (resting heart rate)		Physical activity		
[66]	Age, weight	Health status (health condition)		Sedentary behavior (activity, duration, location, history physical activity)		
[82]	Name, sex, birthdate, age, the initial weight before the simulation starts, height	Disease history (systolic blood pressure, hypertension treatment, diabetes illness, smoking habits)	Consumed food items and quantities	Physical activities, duration of these activities	Nutrition and physical activities preferences	
[63]	Gender, age	Disease history (smoking			User message	

		habits quitting date, cigarette consumption, weekly average expenditure in cigarettes, standardized nicotine dependence, standardized motivation to quit)			rating, 51 extra meta-features questionnaire (patients' comorbidities, living companions, physical activity frequency, I-change model's key factors)	
[64]	Demographic	Health status (clinical information)			Patient ratings, questionnaire (patients' statement of initial interest)	App interaction (sections of the app on the same topic as the messages or re-read messages on a given topic)
[65]					Patient ratings of motivational influences of messages	Patient website visits after viewing messages (frequency at which message promoted engagement, characteristics of the smokers that received these

						messages)
[46]					Individual factors, beliefs, outcome expectations, and social factors	
[83]		Medical conditions			Needs and requirements	
[47]					Therapy task ratings	Users context
[70]		Diagnosed disease			User reviews	
[71]					Medical consultation text	
[26]		Blood pressure		Wearable activity trackers		
[84]	Capability , socioeconomic status	Health condition			preference	
[85]		Electronic health record			Physical activity trackers	electronic subscription, browsing behavior
[32]	Learning capabilities	Patient's condition, treatment				
[72]		Medical data				
[30]		Heart rate variability measured by Bluetooth ECG		Physical activity level measured by Fitbit activity tracker	Recovery, distress, sleep quality, fatigue perceptions through in-app	

					questionnaires	
[73]					Ratings given by the patients	
[56]		Disease severity, allergies		Total energy required for daily preferences		
