# Supplementary material for: Health Recommender Systems Development, Usage, and Evaluation from 2010 to 2022: A Scoping Review
Source: Int J Environ Res Public Health. 2022 Nov 16;19(22):15115. doi: 10.3390/ijerph192215115 (PMC9690602; doi:10.3390/ijerph192215115)
Supplement: Supplementary file 1 [file ijerph-19-15115-s001.zip › Supplement 4.pdf]

Supplement S4

Table S9. Other health recommender system reviews.

Title	Database, timespan	Searching keywords	Summary of included studies	Difference from our review
[12] Recommender systems in mobile apps for health a systematic review	ACM, IEEE, Springer, Science Direct,<br>04-08 of April 2016	(health OR healthcare OR “health care” OR “health-care” OR “M-health” OR ehealth OR “smart health”) AND (“recommender system” OR “recommendation system”) AND (mobile OR smartphone OR tablet)	1) Application area<br>2) What is recommended?<br>3) How is it recommended?<br>4) Device, Operation Systems	1) Only focused on mobile-based HRS.<br>2) No user model.<br>3) No system evaluation.
[13] User models for personalized physical activity interventions: scoping review.	PsycINFO, PubMed, Scopus, Web of Science, 2013-August 23, 2018	(fitness OR exercise OR “physical activity” OR “activity level” OR “active living”) AND (intervention OR recommend* OR prescribe OR prescription<br>OR feedback OR message) AND (tailor* OR personaliz* OR personalis*) AND (mobile OR internet OR computer OR device OR “fitness trackers” OR website OR online) AND (profil* OR model)	1) Overview of studies (objective, intervention presentation, intervention generation, intervention generation technique, theoretical model, personalization, user models)<br>2) User model parameters (physical activity profile, demographics, medical data, behavior change technique parameters, contextual information)<br>3) Results of individual studies – randomized controlled trials (dataset size, variables evaluated, results)	1) Only focused on papers about physical activity.
[14] Analyzing	ScienceDirect,	((“recommender systems”) OR	1) Domain (therapeutic area, target	This review included

recommender systems for health promotion using a multidisciplinary taxonomy: A scoping review	PsycInfo, ACM, IEEE, PubMed, January 1, 2007, to October 18, 2016	(“recommender system”) OR (“recommendation systems”) OR (“recommendation system”)) AND (health OR patient OR patients).	population, type of recommendation, device interface, tailoring, country);<br>2) Methodology and procedures (used metrics to assess performance, number of tested users, effectiveness on patients, success percentage, duration of the total intervention, number of sessions, electronic health record connection, cost effectiveness);<br>3) Health promotion theoretical factors and behavior change theories (attitude, social influence, self-efficacy, action and coping planning, supporting identity change, rewarding abstinence, advising on changing routines, advising on medication use)<br>4) Technical aspects (recommendation interface, recommendation technology, finding recommendations, initial profile generation technique, profile representation technique, profile learning technique, relevance feedback, profile adaptation technique, information filtering method, user-profile item matching technique)	studies 1) with or without user models, 2) with or without personalization.
[15] Combined interventions for physical activity, sleep, and diet using smartphone apps: A	PubMed, ScienceDirecy, PsycINFO, January 2015 --- April 17	((food OR diet) AND (physical activity OR exercise) AND Sleep AND (mobile intervention OR smartphone app) AND (personalized	Summary of included studies (design, duration, sample, app, accompanying device, aim, intervention, personalization, behavioral theory, user profile, personalization	1) This review included personalization generated automatically,

scoping literature review	2018	recommendation OR adaptive feedback)).	parameters, outcomes, measures, results)	manually, and semi-automatically.<br>2) No recommendation technologies
[16] Personalization in real-time physical activity coaching using mobile applications: A scoping review	Pubmed, ACM, ScienceDirect, IEEEexplore, PsycINFO, CINAHL, UCDlibrary;<br>2007 --- 2018	(personalized OR personalised OR personalization OR personalisation OR individualized OR individualised OR individualization OR individualisation OR tailored OR tailoring) AND ("physical activity" OR "daily activity"<br>OR walking OR exercise OR exercising OR "activities of daily living") AND (coach OR coaching OR feedback OR motivate<br>OR motivation OR stimulate OR stimulation OR promote OR promotion) AND (app OR application OR system OR device)	1) General system characteristics (system, studies, target population, target activity, summary, platform, market availability);<br>2) Coaching type, system, personalization (feedback, goal setting, user targeting, inter-human interaction, self-learning, context awareness, adaptation);<br>3) Behavior change foundation & gamification;<br>4) System evaluation methods (system, evaluation studies references, study design, population, study description, outcomes measures, persuasive strategies comp.)	1) No user models.<br>2) No recommendation technologies.
[17] Health recommender systems: A state-of-the-art review	IEEE, ACM, Science Direct, Springer Link, PubMed<br>January 2006 --- august 2018	health recommender system, recommendation system, health social network.	1) Method (content based, collaborative filtering, hybrid based);<br>2) Technique (the way in which the relationships between items are computed);<br>3) Recommendation area (wellness, diagnosis and medication, healthcare services, medical resources);	1) No user model.<br>2) No recommended item.<br>3) No System evaluation.

			4) Knowledge representation (natural language, attribute-value pairs, ontologies, feature vectors fuzzy set)	
[18] A systematic review of tailored eHealth interventions for weight loss	EBSCO, Science Direct, PubMed, EMBASE, Web of Science, Till 15 March 2018	tailoring AND eHealth AND overweight	1) Characteristics (study aim, country, population, intervention, comparison, outcomes, study design);<br>2) Tailoring methods (theory stated and behavioral principles, when and how tailoring was conducted, tailored output, type and mode of delivery, engagement).	1) Only focused on weight-loss research.<br>2) No recommendation technologies.
[19] Health Recommender systems: Systematic Review	ACM digital library, IEEE Xplore, PubMed, No time limit	(recommender OR recommendation systems OR recommendation system) AND (health OR healthcare OR patient OR patients)	1) HRSs recommended items;<br>2) Recommender techniques;<br>3)System evaluation;<br>4) Interface and Transparency	1) no user model;<br>2) no health domain
[20] A Systematic Literature Review on Health Recommender Systems.	Sciencedirect, IEEE, Scopus,<br>2002-2012	"health", "recommendation", "system", "recommender", "eHealth"	1) aim of the paper<br>2) recommendation methods<br>3) Pros and Cons	1) no user model<br>2) no system evaluation<br>3) no health domain

References

12. Ferretto LR, Cervi CR, de Marchi ACB. Recommender systems in mobile apps for health a systematic review. In: *2017 12th Iberian Conference on Information Systems and Technologies (CISTI)*; June 21-24, 2017: 1-6; Lisbon, Portugal.

13. Ghanvatkar S, Kankanhalli A, Rajan V. User models for personalized physical activity interventions: scoping review. *JMIR mHealth and uHealth* 2019; 7(1): e11098.

14. Hors-Fraile S, Rivera-Romero O, Schneider F et al. Analyzing recommender systems for health promotion using a multidisciplinary taxonomy: A scoping review.

Int J Med Inform 2018; 114: 143-155.
15. Kankanhalli A, Saxena M, Wadhwa B. Combined interventions for physical activity, sleep, and diet using smartphone apps: A scoping literature review. *Int J Med Inform* 2019; 123: 54-67.
16. Monteiro-Guerra F, Rivera-Romero O, Fernandez-Luque L et al. Personalization in real-time physical activity coaching using mobile applications: a scoping review. *IEEE J Biomed Health Inform* 2019; 24(6): 1738-1751.
17. Pincay J, Terán L, Portmann E. Health recommender systems: a state-of-the-art review. *In: 2019 Sixth International Conference on eDemocracy & eGovernment (ICEDEG)*; April 24-26, 2019: 47-55; Quito, Ecuador.
18. Ryan K, Dockray S, Linehan C. A systematic review of tailored eHealth interventions for weight loss. *Digital health* 2019; 5: 2055207619826685.
19. De Croon R, Van Houdt L, Htun N N, et al. Health Recommender Systems: Systematic Review. *Journal of Medical Internet Research*, 2021, 23(6): e18035.
20. Sezgin E, Özkan S. A Systematic Literature Review on Health Recommender Systems. *In: E-Health and Bioengineering Conference (EHB).: IEEE*; 2013 Presented at: EHB'13; November 21-23, 2013; Iasi, Romania p. 1.
